# Rapid warming and salinity changes in the Gulf of Maine alter surface ocean carbonate parameters and hide ocean acidification

**DOI:** 10.1007/s10533-018-0505-3

**Published:** 2018-10-12

**Authors:** Joseph E. Salisbury, Bror F. Jönsson

**Affiliations:** Ocean Process Analysis Laboratory, University of New Hampshire, Durham, NH 03824 USA

**Keywords:** Gulf of Maine, Ocean acidification, Events, Global warming

## Abstract

A profound warming event in the Gulf of Maine during the last decade has caused sea surface temperatures to rise to levels exceeding any earlier observations recorded in the region over the last 150 years. This event dramatically affected CO_2_ solubility and, in turn, the status of the sea surface carbonate system. When combined with the concomitant increase in sea surface salinity and assumed rapid equilibration of carbon dioxide across the air sea interface, thermodynamic forcing partially mitigated the effects of ocean acidification for pH, while raising the saturation index of aragonite ($\varOmega_{AR}$) by an average of 0.14 U. Although the recent event is categorically extreme, we find that carbonate system parameters also respond to interannual and decadal variability in temperature and salinity, and that such phenomena can mask the expression of ocean acidification caused by increasing atmospheric carbon dioxide. An analysis of a 34-year salinity and SST time series (1981–2014) shows instances of 5–10 years anomalies in temperature and salinity that perturb the carbonate system to an extent greater than that expected from ocean acidification. Because such conditions are not uncommon in our time series, it is critical to understand processes controlling the carbonate system and how ecosystems with calcifying organisms respond to its rapidly changing conditions. It is also imperative that regional and global models used to estimate carbonate system trends carefully resolve variations in the physical processes that control CO_2_ concentrations in the surface ocean on timescales from episodic events to decades and longer.

## Introduction

Global ocean acidification (OA) proceeds as rising CO_2_ levels in the atmosphere (${\text{CO}}_{2({\rm atm})}$) lead to higher oceanic carbon dioxide concentrations via uptake across the air–sea interface. In surface ocean chemistry, the term “carbonate system” refers to a combination of species produced by the equilibria

1 $$ {\text{CO}}_{2} \longleftrightarrow {\text{H}}_{2}{\text{CO}}_{3} \longleftrightarrow {\text{HCO}}_{3}^{-} \longleftrightarrow {\text{CO}}_{3}^{2-} $$

The uptake of atmospheric carbon over time perturbs the carbonate system such that there is an increase in surface ocean CO_2_ (${\text{CO}}_{2({\rm aq})}$) with a concomitant reduction in surface pH. Since the beginning of the industrial revolution, the world’s surface ocean has decreased by about 0.1 pH units (Doney et al. 2009), and further reductions on the order of 0.2–0.3 pH units are expected by 2100 (Feely et al. 2009). Reduction in surface ocean pH due to increasing ${\text{CO}}_{2({\rm atm})}$ is not the only effect on the carbonate system; additionally, OA causes reductions in carbonate ion (${\text{CO}}_{3}^{2-}$) concentration and in the saturation states of calcium carbonate minerals ($\varOmega$) (Bates et al. 2014). While there is still debate on the direct role that ${\text{CO}}_{3}^{2-}$ availability and its proxy $\varOmega$ play in shell development (Bach 2015; Jokiel 2016; Bednaršek et al. 2016), it is widely stated that reductions in ${\text{CO}}_{3}^{2-}$ and $\varOmega$ represent a stressor to a variety of marine invertebrates that fix their shells or skeletons from calcium carbonate (e.g. Waldbusser et al. 2015). How these chemical changes will propagate into marine ecosystems is a subject of growing concern.

The potential threat by OA on marine organisms is of critical importance in the Gulf of Maine (GOM), where much of the value from fishery landings originates from potentially susceptible organisms such as lobsters (*Homarus americanus*) and sea scallops (*Argopecten irradians*), (Cooley and Kite-Powell 2009; Ekstrom et al. 2015). Declining pH, ${\text{CO}}_{3}^{2-}$ concentrations, and the saturation state of the shell building mineral aragonite ($\varOmega_{AR}$), can affect organisms in a variety of ways. The most cited impacts accrue through decreased rates of calcification, particularly during the larval phases of growth (e.g. Barton et al. 2012; Waldbusser et al. 2013), or via increased respiration that can consume energy required for mobility or reproduction (Gledhill et al. 2015). OA can also affect an organism’s immune response, organ development, and olfactory discrimination (Ekstrom et al. 2015; Munday et al. 2009). Despite this knowledge, the effects of OA on individual species and community ecology are not well understood. While a number of studies have been initiated to fill this knowledge void, much of the work continues to be done within the context of controlled experimental studies rather than within functioning ecosystems. As such, it is difficult to assess a species’ response to multiple stressors or mitigative factors that may occur in the natural environment (Breitberg et al. 2015).

In addition to OA, the carbonate system in coastal waters is affected by varying fluxes of Dissolved Inorganic Carbon $(DIC: \sum ({\text{CO}}_{2({\rm aq})}+ {\text{HCO}}_{3}^{-} + {\text{CO}}_{3}^{2-}))$, total alkalinity (TA), and nutrients derived from local or remote sources. These processes are collectively known as coastal acidification and include acidic river discharge (Salisbury et al. 2008), atmospheric fluxes of acidic and alkaline compounds occurring predominantly in coastal regions (Doney et al. 2007), and coastal eutrophication. The latter is attributable to land- and atmospherically-derived nutrient fluxes that promote intense autotrophic production with subsequent ${\text{CO}}_{2({\rm aq})}$ evolution and pH reduction via heterotrophic respiration (Cai et al. 2011).

Physical processes in the GOM (e.g., strong tides, wind-driven mixing, coastal currents) and large annual ranges in sea surface temperature (SST) and salinity generate significant thermodynamic variability in the carbonate system from diurnal to annual timescales. Of particular importance is the pronounced annual cycle of ${\text{CO}}_{2({\rm aq})}$, whereby disequilibrium with the atmosphere is partially balanced by an air sea flux of DIC (Shadwick et al. 2010; Vandemark et al. 2011). The GOM is therefore an ideal region to investigate how the effects of varying SST and salinity on the carbonate system relate to long-term trends driven by OA. In a climatological study using data from 1950 to 2013, the GOM records an annual SST range of $15.5\,^{\circ }{\text{C}}$ and salinity range of 2.2 (Richaud et al. 2016). The annual range alone elicits a significant change in the carbonate system. For example, using approximate mean GOM salinity (32.2), mean TA ($2184\,\upmu {\text{mol\,kg}}^{-1}$) and an atmospherically equilibrated seawater surface of ${\text{pCO}}_{2}$ (presently $\sim 400\,\upmu {\text{atm}}$), temperature alone produces an annual change of 0.013 in ${\rm pH}$ and 1.06 in $\varOmega_{AR}$.

In this work we use simple data-driven decomposition models to explore how the carbonate system is affected by variability in SST, salinity, and its covarying carbonate parameter, TA. We show that over timescales of 5–10 years, such changes can partially mitigate or overwhelm the effects of OA. The variability imposed by thermodynamic changes is described in several texts (e.g. Butler 1991; Stumm and Morgan 1996). All carbonate dissociation and calcite mineral solubility constants are dependent on, and are modeled using temperature, and thus a change in temperature will alter the relative proportions of carbonate species.

Experimental determinations at constant TA, salinity, and DIC show that the sensitivity of the partial pressure of ${\text{CO}}_{2({\rm aq})}$ (${\text{pCO}}_{2({\rm aq})}$ to temperature variability is $4.23 \% \,^{\circ }{\text{C}}^{-1}$, a relationship that is consistent over a majority of the world’s oceans (Takahashi et al. 1993). Similarly, at typical GOM SSTs, assuming no exchange of inorganic carbon with the atmosphere, and a constant mean GOM TA, salinity, and DIC, pH decreases in a nonlinear fashion at a rate of 0.015–0.017 U $^{\circ }{\text{C}}^{-1}$. However given the rapid equilibration timescales indicative of the adjacent Northwestern Atlantic (1–4 months; Jones et al. 2014), in reality the system would lose DIC during warming, which would mitigate the decrease, or even raise pH. For contrast, at constant mean GOM TA, and salinity, with ${\text{pCO}}_{2({\rm aq})}$ held at $400\,\upmu {\text{atm}}$, pH increases slightly ($\sim 0.001$ U $^{\circ }{\text{C}}^{-1}$) due to the release of DIC.

As water temperature increases, the equilibrium in Eq. 1 shifts to favor increases in ${\text{CO}}_{3}^{2-}$ and $\varOmega_{AR}$ (Dickson and Millero 1987). A dependency of $\varOmega_{AR}$ on temperature is also due to the influence of temperature on the apparent solubility product (Ksp). The Ksp for aragonite decreases by $\sim 0.4\%\,^{\circ }{\text{C}}^{-1}$ (Mucci 1983). Over the range of observed GOM SSTs, assuming rapid equilibration, this translates into a $\varOmega_{AR}$ change of 0.05–0.09 U $^{\circ }{\text{C}}^{-1}$. Salinity variability has a more modest effect on the carbonate system of seawater by changing the ionic strength of the solution (Harris 2010), which decreases activity coefficients and, as a result, the values of pH, and $\varOmega_{AR}$. Tracking salinity also enables regional modeling of the quasi-conservative TA (e.g. Lee et al. 2006; Cai et al. 2010) that can be used as one of two parameters needed to resolve the full carbonate system (Wolf-Gladrow et al. 2007).

Within the GOM, the decade spanning 2005–2014 was characterized by an extreme warming event with average SST increasing by over $0.2\,^{\circ }{\text{C\,y}}^{-1}$. Satellite observations of SST within the GOM show that the region was warming at a faster rate than 99% of the global ocean (Pershing et al. 2015), with the highest average annual values exceeding over 150 years of observations held in NOAA’s Merged Land-Ocean Surface Temperature Analysis database (Vose et al. 2012). The warming initiated ecosystem changes that included a northward (or deeper) shift in the distributions of many planktonic and nektonic organisms as they sought out suitable temperatures (Nye et al. 2009; Fogarty et al. 2012). Enhanced warming was accompanied by an increase in salinity that is consistent with a change in water mass distribution related to a retreat of the Labrador Current and a northerly shift of the Gulf Stream as described in Saba et al. (2016) and Grodsky et al. (2017).

We describe the relationship between physical variability and the carbonate system in the GOM during a time span over which large changes in SST and salinity were observed. Our main emphasis seeks to quantify the effects that recent extreme environmental conditions played in driving changes in ${\rm pH}$ and $\varOmega_{AR}$ and to highlight the difficulties that variability in physical drivers may cause in resolving longer-term trends in OA. The work is structured as follows. “Description of study region, methods, and data” section describes the observations of SST, salinity, and carbonate parameters in the GOM, as well as the model framework and algorithms used to determine carbonate system variability. “Results” section examines the causes of variability in the carbonate parameters by decomposing the model into components that highlight atmospheric versus physical influences. We discuss the implications of the recent heating event and sub-decadal variability in terms of potential ecosystem stress and the longer-term trends in OA in “Discussion” section.

**Fig. 1:**
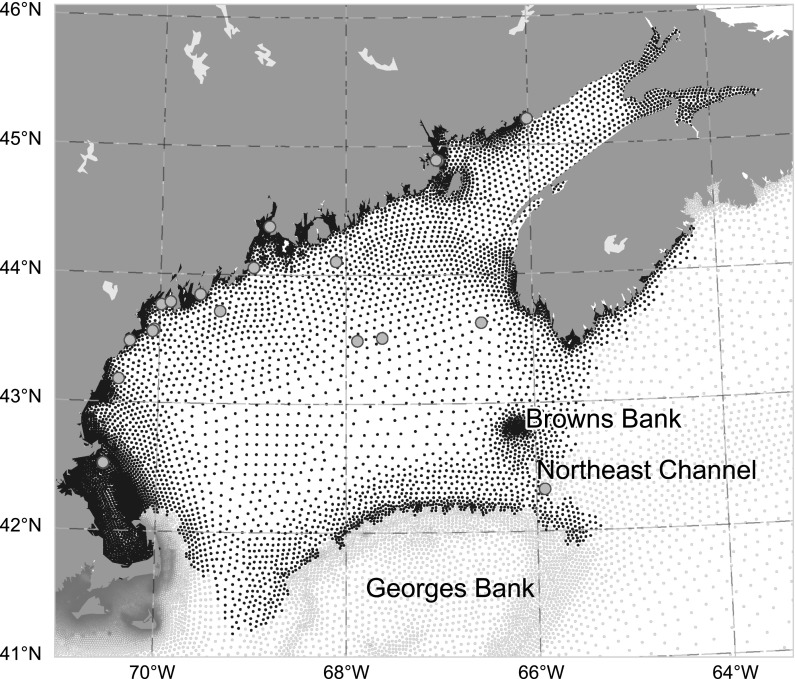
Region map and grid of the model used in this study. The Gulf of Maine domain is shown as black grid nodes and periphery areas as red. Locations of present or former NERACOOS buoys used for validation shown in green. (Color figure online)

## Description of study region, methods, and data

### Site description

The GOM is a productive temperate continental shelf sea bounded by Cape Cod to the south and Nova Scotia to the northeast (Fig. 1). It is well known for its large semidiurnal tides and their resulting impact on mixing, and also for the high commercial value of its fish and shellfish landings. It is separated from the open northwest Atlantic by both Georges and Browns Banks. Considerable control on seasonal to inter-annual circulation patterns is exerted via shelf-sea exchange through the northheast Channel (NEC) which separates Georges and Browns Banks from the fresher coastal source waters on the Scotian Shelf (Townsend 1991; Pringle 2006; Hetland and Signell 2005; Geyer et al. 2004; Feng et al. 2016). The GOM has an average tidal range $>3.0\,{\text{m}}$, and experiences large seasonal amplitudes in surface salinity (Geyer et al. 2004), net primary productivity (O’Reilly et al. 1987), SST and ${\text{pCO}}_{2({\rm aq})}$ (Vandemark et al. 2011). The key circulation feature impinging on the region is the Maine Coastal Current (MCC), which flows counterclockwise and delivers freshwater and constituents from the northeast along the coast and into the Gulf (Pettigrew et al. 2005).

### Data preparation

We base our study on a combination of data sources that include output from a physical General Circulation Model (GCM) for salinity; satellite-derived SST and chlorophyll, and ${\text{pCO}}_{2({\rm aq})}$ observed in the GOM. The carbonate system is modeled using thermodynamic equilibrium equations as described in the Handbook of Methods for Analysis of the Various Parameters of the Carbon Dioxide System in Seawater (Dickson and Goyet 1994):

2 $$ {[}{CO}_2*] + [H_2O] \Longleftrightarrow [H^+] + [{HCO}_3^-] \Longleftrightarrow [H^+] + [{CO}_3^{2-}], $$

with the apparent dissociation constants

3 $$ K1= \frac{[H^+] [{HCO}_3^-]}{[{\text{CO}}_{2({\rm aq})}*]} \quad {\text{and}} \quad K2 = \frac{[H^+] [{CO}_3^{2-}]}{[{HCO}_3^-]}, $$

where brackets represent the stoichiometric concentrations of the chemical species and [${\text{CO}}_{2({\rm aq})}$*] represents the sum of the combined concentrations of ${\text{CO}}_{2({\rm aq})}$ and ${\rm H}_2{\rm CO}_3$.

A full description of the carbonate system requires salinity, temperature, pressure, and two carbonate parameters—in this study TA and ${\text{pCO}}_{2({\rm aq})}$. Total scale pH and $\varOmega_{AR}$ are subsequently estimated using the CO2SYS package (Lewis et al. 1998). The K1 and K2 constants are based on Mehrbach et al. (1973) and refitted by Dickson and Millero (1987), with the borate-to-salinity ratio of Uppström (1974). Alkalinity modifications by phosphate and silicate are assumed to be negligible. The saturation state with respect to aragonite is defined as

4 $$\varOmega_{Ar} = \frac{[Ca^{2+}][CO_3^{2-}]}{K_{sp}} $$

where ${\rm Ca}^{{2+}}$ and ${\text{CO}}_{3}^{2-}$ are the molar concentrations of calcium and carbonate ions in solution, and K$_{sp}$ is the solubility product of the mineral aragonite that is modeled as a function of in situ temperature, salinity, and pressure.

### Data and algorithms

Our analyses are performed on time series data that begin in September 1981 and run through December 2014. All spatially resolved datasets are averaged over the GOM domain to generate integrated system-wide time series with a monthly temporal resolution. SST is based on the $1/4^{\circ }$ daily Optimum Interpolation Sea Surface Temperature Version 2 (dOISSTv2), which combines observations from satellites, ships, and buoys into a blended product on a unified grid. The main data sources are NOAA’s Advanced Very High Resolution Radiometer (AVHRR) 7–19 satellites. The resulting product typically has an average root-mean-square error (RMSE) of $0.3\,^{\circ }{\text{C}}$ compared to buoy data (Banzon et al. 2016; Reynolds and Chelton 2010).

Salinity is derived from the Northeast Coastal Ocean Forecast System (NECOFS). NECOFS is an integrated atmosphere-ocean model forecast system designed for the Northeast US coastal region covering a computational domain from the southern part of Long Island Sound to the northern part of the Scotian Shelf (Li et al. 2017). The system includes the mesoscale Weather Research and Forecasting model (WRF) and the unstructured grid Finite-Volume Community Ocean Model (FVCOM-GOM, Chen et al. 2006) GCM. The FVCOM-GOM grid covers the GOM region and is enclosed by an open boundary running from the Delaware Shelf to the Scotian Shelf. The horizontal grid has a resolution (measured by the length of the longest edge of a triangular cell) that varies from 0.3 to 15 km over the entire domain. The resolution in the Bay of Fundy ranges from 0.5 km inside inlets to 2.0 km along the coast and 4.0 km in the interior of the Bay. NECOFS is a product of the Northeast Regional Coastal Ocean Observation System (NERACOOS), with support from the Massachusetts Fishery Institution and the MIT Sea Grant Program. The salinity time series is validated using data from five NERACOOS moorings in the GOM (Fig. 1). We use monthly averages for each buoy and perform a point-to-point comparison with each nearest grid cell in the model. We note that uncertainty of salinity (Table 1) can be considered conservative due to spatial and temporal mismatches between buoy and modeled data.

**Fig. 2:**
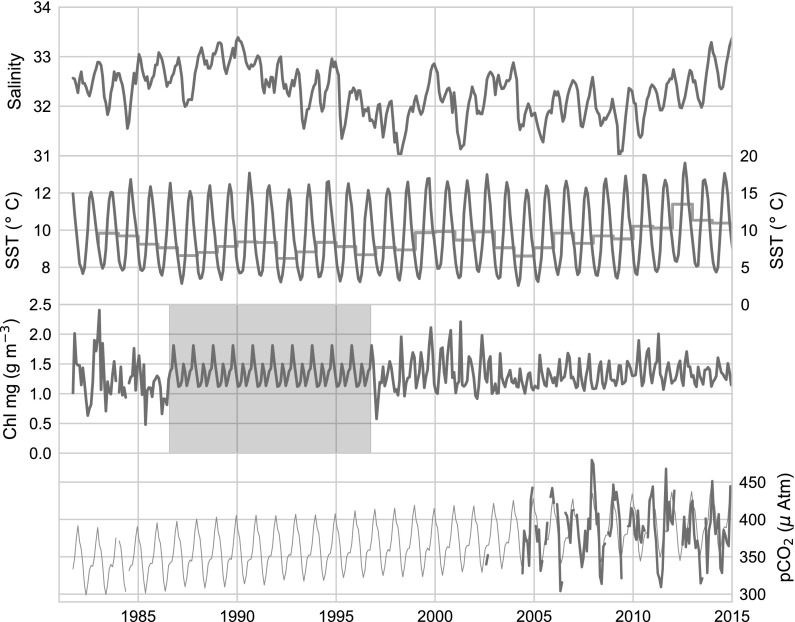
System-wide climatologies of time series data used for model development. Top panel: salinity from FVCOM; second panel: SST from dOISSTv2, the red curve shows annual averages; third panel: satellite derived Chl, the grey box highlights the time period when no ocean color sensors were available and monthly climatology from 3 years before and after fills the gap; bottom panel: monthly mean values of the SOCAT V5 database in the GOM domain (blue), underlain by ${\text{pCO}}_{2({\rm aq})}$ model output described in the text (green). (Color figure online)

Time series of chlorophyll (Chl) are made using data from the NASA satellites’ Coastal Zone Color Scanner (CZCS 1981–1986, NASA Goddard Space Flight Center 2014a), the Ocean Color-Temperature Satellite (OCTS 1996, NASA Goddard Space Flight Center 2014c), the Sea-Viewing Wide Field-of-View Sensor (SeaWiFS 1997–2002, NASA Goddard Space Flight Center 2014d), and the Moderate Resolution Imaging Spectroradiometer (MODIS-Aqua 2002–present, NASA Goddard Space Flight Center 2014b). 4 km Level 3 mapped monthly-mean fields from the processing version 2014.0.1QL are matched to the FVCOM grid by identifying the satellite grid cell with which each node in the FVCOM grid overlaps. Time gaps between CZCS and OCTS, and OCTS and SeaWiFS, are filled by using a monthly climatology based on the three previous years before and the 3 years after the gap (Fig. 2, third panel from top).

Zonal averages ($40^{\circ }{\text{N}}$–$50^{\circ }{\text{N}}$) of marine atmospheric boundary layer mole fraction of CO_2_ (${\text{xCO}}_{2}$) from 1981 to 2014 were acquired from the NOAA Earth System Research Laboratory GLOBALVIEW-CO2 database (Masarie and Tans 1995). Atmospheric CO_2_ partial pressure (${\text{pCO}}_{2({\rm atm})}$) was estimated as ${\text{pCO}}_{2({\rm atm})} = {\text{XCO}}_{2}$ (SLP-${\text{pH}}_{2}{\text{O}}$). Sea level pressure (SLP) was set to 1 standard atmosphere (1013.25mb), and water vapor pressure (${\text{pH}}_{2}{\text{O}}$) was calculated as a function of mean monthly GOM SST according to the empirical formula described by Cooper et al. (1998).

TA is modeled as a function of salinity and dOISSTv2 temperature data according to the North Atlantic model of Lee et al. (2006, Table 1, Fig. 2). Model output was compared to all surface TA data found in the National Centers for Environmental Information (${\text{n}}=2140$) producing an RMSE of $11.4\,\upmu {\text{mol\,kg}}^{-1}$ (Table 1).

### ${\text{pCO}}_{2({\rm aq})}$ algorithm

${\text{pCO}}_{2({\rm aq})}$ is modeled by deriving a relationship between SST, salinity, Chl, day of year, and observed ${\text{pCO}}_{2({\rm aq})}$ based on a modification of methods presented by Signorini et al. (2013). The Signorini et al. algorithm performed reasonably well with modeled ${\text{CO}}_{2({\rm aq})}$ showing an RSME $34.6\,\upmu {\text{atm}}$ for the GOM region. Our algorithm is a multiple linear regression of a similar form, i.e.:

5 $$ pCO_2 = f\left( yd', \left[ K_0-\overline{K_0}\right] , \left[ log_{10}(Chl)-log_{10}(\overline{Chl})\right] \right) + slope (year-2004) $$

where $K_0$ is the solubility for ${\text{CO}}_{2({\rm aq})}$ and *Chl* is satellite chlorophyll. $\overline{K_0}$ and $\overline{Chl}$ are system-wide mean values for the data set. $yd'$ is the fit of a third order Fourier function that models a daily climatology of ${\text{GOM\,pCO}}_{2({\rm aq})}$ based on day of year. The *slope* term represents the annual derivative of the atmospheric ${\text{pCO}}_{2({\rm aq})}$ estimates for the North Atlantic region. These data have been smoothed by 3 months to account for the estimated time required for ${\text{CO}}_{2({\rm aq})}$ to equilibrate into the surface ocean in our region (Jones et al. 2014).

Training data for the multi regression model of ${\text{pCO}}_{2({\rm aq})}$ are taken from the Surface Ocean ${\text{CO}}_{2({\rm aq})}$ Atlas (Bakker et al. 2016) within our domain. In an effort to avoid extreme ${\text{pCO}}_{2({\rm aq})}$ values found in regional river plumes (Cai et al. 2010; Salisbury et al. 2009), we remove all ${\text{pCO}}_{2({\rm aq})}$ data measured at salinities $<30.5$. Further, to avoid disproportionate effects from extreme outliers, we remove data below and above the 1 and 99 percentiles respectively. The resulting data set contained 200 784 values. These data were averaged by month prior to regression analysis. Model coefficients, results, and uncertainty are found in Fig. 2 and Table 1.

Our approach differs from that of Signorini et al. (2013) in three ways. First, we substitute SST and salinity with $K_0$, a function of temperature, salinity, and pressure (Weiss 1974). This approach provides a better linear relationship compared to the non-linearity that changing temperature imposes on ${\text{CO}}_{2({\rm aq})}$ solubility. Second, instead of a simple sine function for day of year, we use $yd'$, which represents a better approximation of the annual ${\text{pCO}}_{2({\rm aq})}$ cycle. Finally, we base the trend in ${\text{CO}}_{2({\rm atm})}$ on observations instead of the linear increase of $1.68\,\upmu {\text{atm\,y}}^{-1}$ used in Signorini et al. (2013). While the average linear slope determined here is similar ($1.78\,\upmu {\text{atm\,y}}^{-1}$), our approach accounts for the non-linear increase in ${\text{CO}}_{2({\rm atm})}$ over time (Fay and Mckinley 2013).

**Table 1:** Uncertainty of modeled parameters

Parameter	Uncertainty	Data sources
SST	$0.3\,^{\circ }{\text{C}}$	Banzon et al. (2016) and Reynolds and Chelton (2010)
Salinity	0.49	“ GOM buoys vs FVCOM model fields” section
TA	$11.4\,\upmu {\text{mol\,kg}}^{-1}$	“Unpublished observations vs Lee model” section
${\text{pCO}}_{2({\rm aq})}$	$20.7\,\upmu {\text{atm}}$	“SOCAT data vs modified Signorini model” section
$\varOmega_{AR}$	0.070	“Monte Carlo simulation” section
${\rm pH}$	0.015	“Monte Carlo simulation” section

Uncertainties are measured as root mean square error (RMSE) defined as $\frac{ \sum_{i=1}^{n}\left( y-x \right)^2}{n}$ , where *x* are observations, *y* model estimates, and *n* the number of observations

### Monte Carlo simulations

The uncertainties of $\varOmega_{AR}$ and ${\rm pH}$ are estimated via 10,000 Monte Carlo simulations using SST, salinity, TA, and ${\text{pCO}}_{2({\rm aq})}$ data with accompanying uncertainty (standard deviation) values (Table 1). Prior to analysis, single-sample Kolmogorov–Smirnov tests were performed on each variable to verify the data were normally distributed.

### Anomalies and sensitivity analysis

To better elucidate long-term trends and short-term variability obscured by the seasonal cycle, we use 2004 as a reference to which we compare all other data. This year is noteworthy, as it represents the beginning of the recent extreme warming event (Pershing et al. 2015). The modeled time series of TA, ${\text{pCO}}_{2({\rm aq})}$, salinity, and SST are used to construct time series of $\varOmega_{AR}$ and ${\rm pH}$. Anomalies are calculated by removing mean monthly values in 2004 from all other years, month by month. We use the resulting time series to estimate the sensitivity of $\varOmega_{AR}$ and ${\rm pH}$ to variability imposed by OA, the effects of variable TA, and the combined effects of SST and salinity variability, the latter which are intended to track the variability of carbon dioxide solubility. For this work, we narrowly define OA as the changes to the carbonate system brought about by the increase in sea surface ${\text{pCO}}_{2({\rm aq})}$ that are directly attributable to increasing ${\text{CO}}_{2({\rm atm})}$.

To understand the relative contribution of changes in ${\text{pCO}}_{2({\rm aq})}$, TA and combined SST and salinity on ${\rm pH}$ and $\varOmega_{AR}$ variability, we perform the decompositions shown in Eqs. 6 and 7. The partial derivatives quantify changes in ${\rm pH}$ and $\varOmega_{AR}$ attributable to incremental changes in ${\text{pCO}}_{2({\rm aq})}$, TA, and $\varPi$, the last, which represents the combined temperature and salinity effects intended to track changes in ${\text{CO}}_{2({\rm aq})}$ solubility (Eq. 8), with $\zeta$ representing the parameter of interest (i.e. ${\rm pH}$ or $\varOmega_{AR}$).

6 $$ \varDelta [pH]=  \frac{\delta pH}{\delta pCO_2} \varDelta pCO_2 + \frac{\delta pH}{\delta TA} \varDelta TA + \varPi $$

7 $$ \varDelta [\varOmega_{AR}]=  \frac{\delta \varOmega_{AR}}{\delta pCO_2}\varDelta pCO_2 + \frac{\delta \varOmega_{AR}}{\delta TA} \varDelta TA + \varPi $$

8 $$ \varPi= \left[ \frac{\delta \xi }{\delta SST} \varDelta SST + \frac{\delta \xi }{\delta Salinity} \varDelta Salinity \right] $$

The sensitivity to each variable is calculated by holding each of the other components constant (Table 2). The constant values are based on mean data for each month of our reference year, 2004. Our approach produces monthly time series showing the relative contributions of ocean acidification, variable TA and the combined effects of variable SST and salinity, referenced to each month of 2004. We investigate the behavior of OA on $\varOmega_{AR}$ and ${\rm pH}$ (Table 2) by imposing the atmospheric ${\text{pCO}}_{2({\rm atm})}$ smoothed by 3 months, to account for ${\text{CO}}_{2({\rm aq})}$ equilibration.

**Table 2:** Description of sensitivity analyses

Process	Modeling concept	Description
Combined SST and SSS effects ($\varPi$)	TA and ${\text{pCO}}_{2({\rm aq})}$ held constant	Response caused by heat flux, net freshwater flux and ocean circulation
TA effects	SSS, SST, and ${\text{pCO}}_{2({\rm aq})}$ held constant	Response caused from changes in TA
Ocean acidification effects	Salinity, SST, and TA held constant; ${\text{pCO}}_{2({\rm aq})}$ increases proportional to atmospheric values	Response from increasing ${\text{CO}}_{2({\rm atm})}$ equilibrating into surface water

First column, process examined; second column, model method during sensitivity analyses; third column, description of process examined

## Results

### The annual cycle of carbonate parameters

**Fig. 3:**
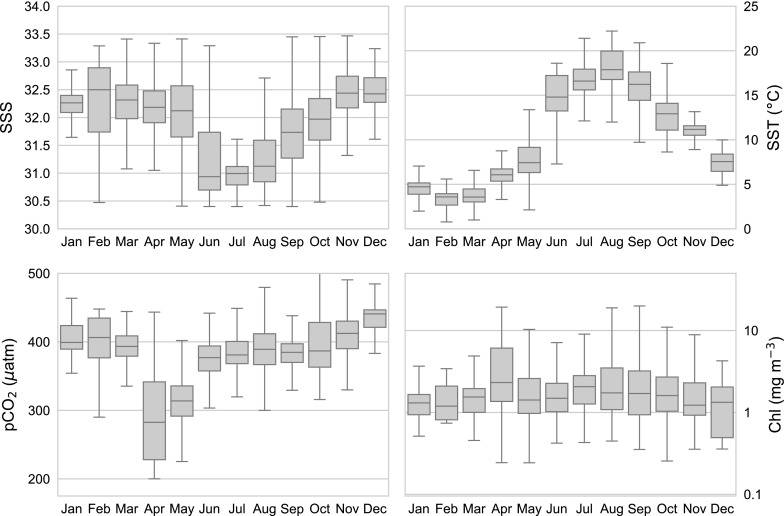
Annual cycles of data used for model parameterization. Boxplots show median value (white dot), 25th and 75th percentiles (black bar) and range of data (vertical line), with statistical outliers not shown. Salinity (top left), pCO$_{2}$ (top right) and SST (bottom left) taken from the SOCAT V5 database, with log Chl data taken from ocean color sensors coincident with the SOCAT data

Our results are affected by the combination of seasonal cycles, interannual variability, and decadal-scale changes. These different scales are evident in Fig. 2 (e.g. salinity), where full time series of the data used for model parameterization are presented. The seasonal cycles of these variabiles are detailed in Fig. 3, where the upper right panel highlights the annual cycle of SST, ranging from a low of $<4\,^{\circ }{\text{C}}$ in February and March to a mean high of $18\,^{\circ }{\text{C}}$ in August. Salinity (upper left) has a range of $\sim \,1.5$ from a low of 31.0 in July to a high of 32.5 in November. The cycle of satellite Chl (bottom right) shows a peak in April, which corresponds to the maximum rates of net primary production (e.g. Behrenfeld and Falkowski 1997; Friedrichs et al. 2009). While not apparent in the climatology, a smaller, shorter duration, peak corresponding to a fall bloom, is often observed during September or October (e.g. Riley 1947; Townsend and Spinrad 1986; Thomas et al. 2003).

This combination of seasonal variability in salinity, SST, and net production controls much of the annual cycle of ${\text{pCO}}_{2({\rm aq})}$ in the GOM and is largely consistent with the seasonal dynamics described by Vandemark et al. (2011), who have suggested that changes in ${\text{pCO}}_{2({\rm aq})}$ are controlled by the countervailing annual cycles of solubility and net biological process. The biological “new year” begins with an intense spring phytoplankton bloom that typically extends from March to May. By removing DIC, the spring bloom generates the most significant perturbation to ${\text{pCO}}_{2({\rm aq})}$ throughout the year. Following the bloom, surface water warms, promoting an increase in ${\text{pCO}}_{2({\rm aq})}$. Changes in $K_0$ due to the summer warming are expected to increase $\varOmega_{AR}$ and decrease ${\rm pH}$. The annual salinity cycle, which controls TA, imposes a modest modulation of ${\text{pCO}}_{2({\rm aq})}$ via buffering of the carbonate system, leading to slightly higher ${\text{pCO}}_{2({\rm aq})}$ values when the salinity is low and vice versa. A fall bloom may also cause a slight reduction in ${\text{pCO}}_{2({\rm aq})}$ via uptake of DIC. As winter approaches, the water becomes well mixed, entraining deeper waters with higher DIC concentrations to the surface, raising the ${\text{pCO}}_{2({\rm aq})}$, while lowering $\varOmega_{AR}$ and ${\rm pH}$ (Wang et al. 2017).

### Anomaly analyses

**Fig. 4:**
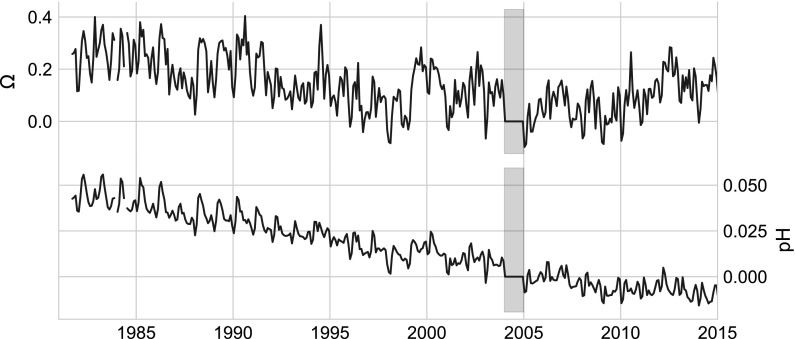
Anomalies relative to monthly 2004 data. $\varOmega_{AR}$, (top panel), and ${\rm pH}$ (bottom panel). Each time series was estimated in CO2SYS using modeled ${\text{pCO}}_{2({\rm aq})}$, TA, SST and salinity. Month by month values for 2004 were subtracted from the time series to produce the anomalies. The grey bar highlights data for 2004, zeroed out by subtraction

Our anomaly analyses (Fig. 4) show that ${\rm pH}$ (lower panel) exhibits a markedly different behavior than $\varOmega_{AR}$ (top panel). ${\rm pH}$ declines at an average rate of $0.0018\,{\text{y}}^{-1}$ from 1981 to 2015, primarily in response to the OA signal imposed on the carbonate system by increasing atmospheric ${\text{pCO}}_{2({\rm aq})}$. The effect of OA on $\varOmega_{AR}$ is partially obscured because of their greater sensitivity to variations in SST and salinity. Both parameters show significant interannual variability caused by differential timing and magnitude of the seasonal cycle in SST, salinity, and net community production relative to 2004. It is notable that $\varOmega_{AR}$ shows a higher interannual variability (often $> 5\%$, $\upmu \varOmega_{AR} = 1.96$) than ${\rm pH}$ ($\ll 1\%$, $\upmu {\rm pH} = 8.06$). The long-term trend in ${\rm pH}$ is not entirely consistent over the full time series, but varies on decadal scales with patterns in the SST and salinity data (see Fig. 4). For example, the time periods 1981–1987, 1995–2001, and 2004–2014 show considerable deviation from the long-term trend 1981–2015 driven by OA (i.e. ${\text{pH}}= -\,0.018$ and $\varOmega_{AR}= -\,0.065\,{\text{per\,decade}}$, Table 3, See OA). The decadal variability is more pronounced for $\varOmega_{AR}$, which is dominated by the variation in TA and combined SST and salinity, with less dependence on the OA signal.

### Sensitivity analyses

**Fig. 5:**
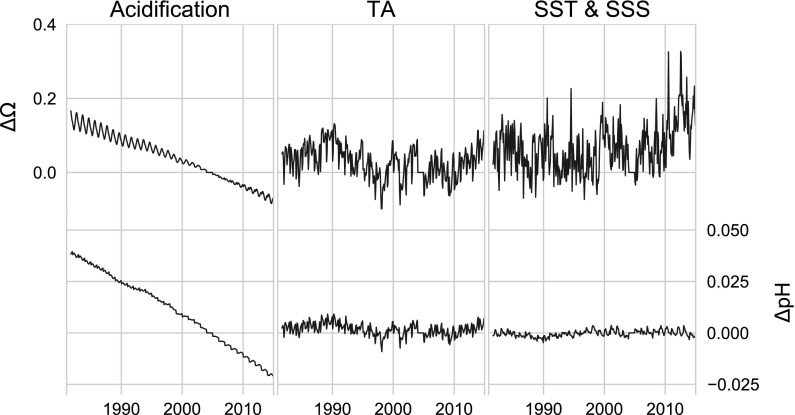
The sensitivity of $\varOmega_{AR}$ (top panels) and ${\rm pH}$ (bottom panels), to OA (left panels), variable TA (center panels) and combined effects of variable SST and salinity (right panels). Slope information for various time intervals can be found in Table 4

By modeling the carbonate system while holding certain terms constant, we are able to investigate in greater detail how specific processes affect carbonate parameters. Figure 5 shows results from these analyses. We note the annual amplitude of each curve is partially affected by our choice to hold variables relative to each month of 2004. While OA imparts a signal into each parameter, the effect is most significant for ${\rm pH}$ (Table 3). The trends in TA perturbations are significant when the entire time series is viewed (Table 3), but are even more pronounced when the data are divided into sub-decadal periods from 1991 to 1997 and 2004 to present, intervals that correspond to salinity anomalies in the GOM (Table 4).

**Table 3:** Data perturbations and resultant decadal change and uncertainty of slope over the entire data range (1981–2014)

Perturbation	Parameter	Average decadal change (confidence interval)
No perturbation	$\varOmega_{AR}$	− 0.049 (± 0.009)
	${\rm pH}$	− 0.018 (± 0.0005)
OA	$\varOmega_{AR}$	− 0.065 (± 0.009)
	${\rm pH}$	− 0.018 (± 0.0021)
TA	$\varOmega_{AR}$	− 0.007 (± 0.004)
	${\rm pH}$	− 0.0006 (± 0.0003)
SST and salinity	$\varOmega_{AR}$	0.025 (± 0.006)
	${\rm pH}$	0.003 (± 0.0012)

Uncertainty is estimated as confidence intervals at ${\text{p}}<0.05$ and presented in parentheses. Note that the decadal change attributable to temperature and salinity variability is positive

**Table 4:** The response of $\varOmega_{AR}$ and ${\rm pH}$ to perturbations by acidification, variable TA, and combined variability of SSS and SST over specific time ranges with high variance in SST and salinity

Time range	Parameter	Decadal change		
		OA	TA	SST and SSS
1981–1987	$\varOmega_{AR}$	− 0.057 (± 0.0187)	− 0.092 (± 0.0611)	0.020 (± 0.0001)
	${\rm pH}$	− 0.016 (± 0.0006)	− 0.002 (± 0.0014)	0.002 (± 0.0005)
1991–1998	$\varOmega_{AR}$	− 0.053 (± 0.0077)	n/a	− 0.115 (± 0.0276)
	${\rm pH}$	− 0.015 (± 0.0006)	0.003 (± 0.0009)	− 0.008 (± 0.0020)
2004–2015	$\varOmega_{AR}$	− 0.070 (± 0.0023)	0.156 (± 0.0305)	0.066 (± 0.0180)
	${\rm pH}$	− 0.020 (± 0.0003)	n/a	0.0037 (± 0.0013)

Confidence intervals are shown in parentheses as estimated at ${\text{p}}<0.05$. n/a indicates that the slope is not significant

The combined effects of SST and salinity on ${\rm pH}$ produce small but significant changes over all timescales considered (Tables 3, 4) with the sign of ${\rm pH}$ change being negative or positive depending primarily on the direction of the SST change. The combined effects of SST and salinity cause large variability in $\varOmega_{AR}$ at all time intervals except for 1991–1998 (Table 4). We note that the curves describing the sensitivity of $\varOmega_{AR}$ to TA and combined salinity and SST share similarities for two reasons. First, the TA values and their corresponding buffering capacity are dependent on salinity and, to a lesser degree, temperature (Lee et al. 2006). Second, as discussed in the introduction, decadal variability in SST and salinity often covary due to the interplay between fresher water entering from the north, and warmer waters from the south, with warming accompanied by an increase in salinity (and alkalinity) and vice versa. Both warming and increased alkalinity will increase $\varOmega_{AR}$.

**Fig. 6:**
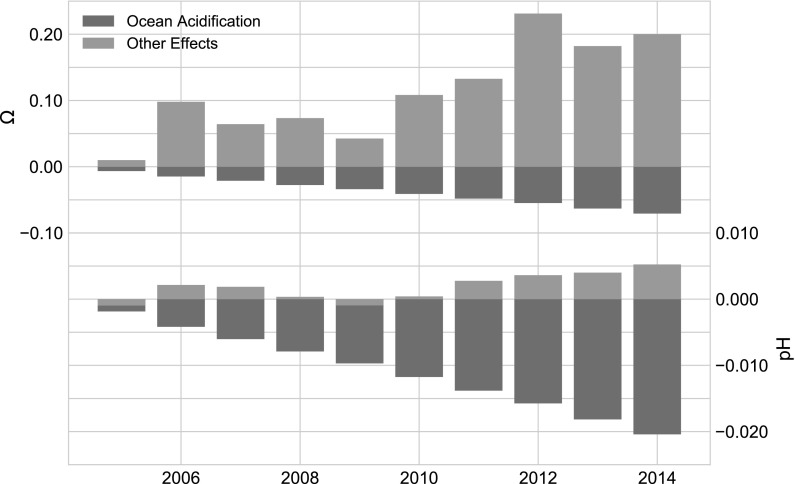
A comparison of ocean acidification effects (blue bars) versus all other effects (green bars) for $\varOmega_{AR}$, (top panel) and ${\rm pH}$ (bottom panel) from 2004 to 2014. Acidification is the dominant cause of variability in pH during this period. However the combination of variable TA, SST and salinity overwhelm the acidification signal for $\varOmega_{AR}$. (Color figure online)

**Fig. 7:**
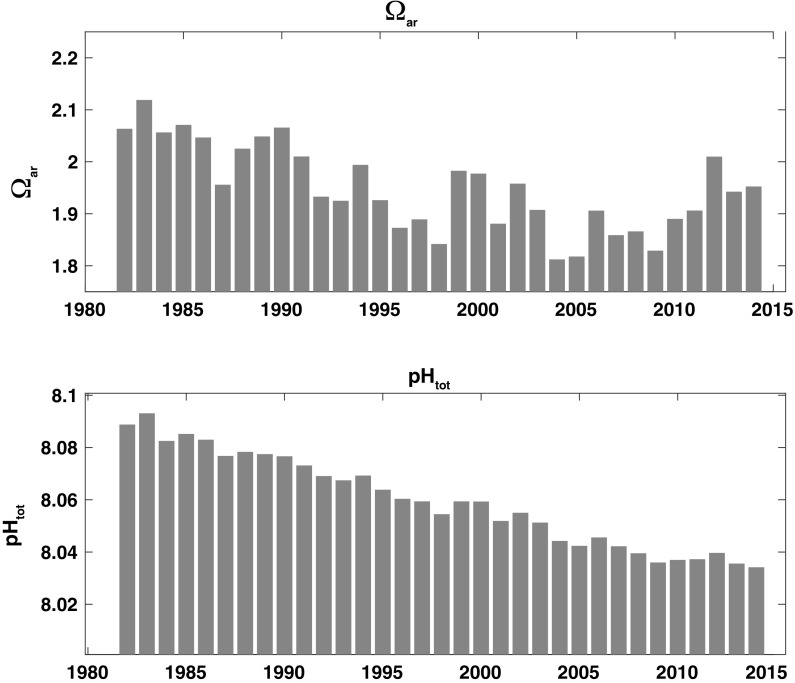
Annual means of $\varOmega_{AR}$, (top panel) and ${\rm pH}$ (bottom panel), intended to demonstrate interannual and decadal scale variability in $\varOmega_{AR}$. Note that data from 1981 have been eliminated since it was not an entire year

Using mean annual values, we assess the respective contribution by acidification and other effects to recent trends in ${\rm pH}$ and $\varOmega_{AR}$. Figure 6 shows a subset of the sensitivity analyses that focus on the extreme warming and salinity event spanning from 2004 to 2014. The figure demonstrates the magnitude of the OA signal relative to all other effects considered here (TA, SST and salinity). During this time OA is clearly the dominating factor affecting the trend in ${\rm pH}$, whereas $\varOmega_{AR}$ is mainly influenced by the other factors (cf. Table 4). These distinctions can also be see in annual mean of the time series in Fig. 7.

## Discussion

Over sufficiently long timescales it is understood that the oceanic carbonate system will respond proportionally to increasing DIC brought about by OA. Several recent studies have presented results showing OA to be the predominant driver of trends in ${\rm pH}$ (Lauvset et al. 2015), ${\text{pCO}}_{2({\rm aq})}$ (Fay and Mckinley 2013; Turi et al. 2016), and $\varOmega_{AR}$ (Jiang et al. 2015). However, trends in these parameters are often found to be larger or smaller than that expected by OA alone. Sensitivity analyses by these investigators demonstrate that processes such as net warming, variable salinity, and upwelling affect the rate of change over time and space. This is particularly true in coastal regions (Turi et al. 2016), where upwelling and freshwater inputs can affect the variability of the carbonate system and increase the length of time series needed to detect statistically distinct differences in the rate of change (Fay and Mckinley 2013; Tjiputra et al. 2014).

Because of the climatological, geographical, and hydrological characteristics found in the GOM and its surrounding watersheds, we expect the OA signal to be modulated by physical forcing. This forcing can occur over sub-daily timescales such as the case in which heat flux or mixing of water masses rapidly change the distribution of carbonate parameters. Figure 6 shows an example occurring at the decadal scale where the OA signal dominates the variability of ${\rm pH}$, but for $\varOmega_{AR}$, the OA signal is overwhelmed by other factors. Over this time period, thermodynamic forcing partially mitigated the effects of OA for ${\rm pH}$, while $\varOmega_{AR}$ increased by an average of 0.14 U. We note that physical forcing leading to a change in surface ${\text{pCO}}_{2({\rm aq})}$ would also alter the disequilibrium of ${\text{pCO}}_{2({\rm aq})}$ with the atmosphere, initiating a change in the rate of the net ${\text{CO}}_{2({\rm aq})}$ flux. Although we could find no published information describing the timescales of mixed layer equilibration of ${\text{CO}}_{2({\rm aq})}$ in the GOM, we assume that it is similar to or less than the adjacent Western Atlantic that exhibits timescales of 1–4 months due to the relatively high gas transfer velocities and moderate mixed layer depths found in the GOM (Jones et al. 2014; Galbraith et al. 2015). Such short equilibrium timescales would mean that increases in surface ${\text{pCO}}_{2({\rm aq})}$ due to warming are accompanied with rapid removal of DIC across the air–sea interface while ${\text{pCO}}_{2({\rm aq})}$ remains higher than ${\text{pCO}}_{2({\rm atm})}$. The opposite would be true during cooling. In the case of heating (increased ${\text{pCO}}_{2({\rm aq})}$), the removal of DIC would raise the ratio of TA:DIC and in turn, modulate the change in ${\rm pH}$ and $\varOmega_{AR}$ to higher values. Thus we speculate that thermodynamic forcing with subsequent gas exchange plays a major role in regulating the status of the carbonate system in the GOM.

Our results demonstrate that ${\rm pH}$ and $\varOmega_{AR}$ in the GOM respond to OA, but are also sensitive to thermodynamic (net heating) and chemical (TA) variability. The magnitude of response to each process is dependent on the parameter and the conditions to which the parameters are subjected. For example, ${\rm pH}$ demonstrates significant correlation with OA over the full time series ($r^2=-\,0.95$, ${\text{p}}<0.001$) (Fig. 4, Table 3). However, ${\rm pH}$ is less correlated with OA during periods with high overall variability (Table 4), owing to greater influences in variability of SST, salinity and TA. $\varOmega_{AR}$ is also affected by OA, but is far more sensitive to relative changes in temperature and salinity than ${\rm pH}$ (Tables 3, 4). Over the entire time range, and for each parameter, the effect on the carbonate systems by TA variability alone generates significant decdal-scale trends (Table 3). Since the distributions of TA closely follow salinity patterns, TA is primarily controlled by decadal scale salinity anomalies (Petrie and Drinkwater 1993) that can be linked to the upstream discharge of the Saint Lawrence River and the behavior of Arctic water masses (Khatiwala et al. 1999), as well as inputs of warm salty slope waters originating from the South (Saba et al. 2016; Townsend et al. 2015). This control has important implications in that discharge from local watersheds to the GOM has been increasing over the last four decades (Huntington et al. 2016; Huntington and Billmire 2014), and with higher precipitation than under current conditions expected in the future (Rawlins et al. 2012), local discharge could continue to increase. The possibility of fresher surface waters in the GOM from both regional and remote sources implies that TA could decrease together with the TA:DIC ratio, and as a consequence, ${\rm pH}$ and $\varOmega_{AR}$ would also decrease.

The sensitivity to temperature shown by $\varOmega_{AR}$ highlights the degree to which interannual warming and cooling events influence longer-term trends. The last decade’s warming and increasing salinity event has had a dramatic effect on $\varOmega_{AR}$ that is $>2.5$ times greater than that of OA alone (Fig. 6, Table 4). Put another way, the changes in salinity and SST linked to the recent event actually served to mitigate the equivalent of 25 years of decline from OA. We note that if this event had instead contributed fresher and cooler waters, the effect would have reversed and greatly exacerbated the impact of OA. While the perturbations in salinity and SST over the last decade are extreme in our data set, smaller SST and salinity fluctuations capable of affecting the consecutive interannual means of $\varOmega_{AR}$ ($> \pm \,5\%$) can be found throughout the time series (Fig. 7). By contrast, such variability is an order of magnitude greater than the interannual variability in the offshore surface waters of the Atlantic and Pacific Oceans (Jiang et al. 2015).

### The hidden signal of ocean acidification

While OA is clearly decreasing $\varOmega_{AR}$ and ${\rm pH}$, it is difficult to observe this signal without sufficiently long time series (Fay and Mckinley 2013; McKinley et al. 2017; Henson et al. 2016). Because the strong seasonality and vigorous physical processes serve to attenuate the OA signal in the GOM, we seek to understand how long a time series of observations would need to be in order to observe the expression of OA. One can evaluate the degree to which total variability obscures the OA signal by estimating the time it would take for the current trend in OA to emerge from the background variability caused by biological and physical processes affecting the carbonate system. One simple approach, termed Time of Emergence (ToE), is designed to detect biogeochemical signals in the context of noise imposed by other natural processes (Keller et al. 2014). ToE is defined as

9 $$ ToE = \frac{2 \sigma }{|S|}, $$

where *S* is the absolute value of the slope of the trend of interest and $\sigma$ is the variance (standard deviation) of the observed time series. Doubling $\sigma$ implies that the signal emerges through twice the observed variability and that ToE will be estimated at the 95% confidence level. Assuming an average positive trend of $1.78\,\upmu {\text{atm\,pCO}}_{2({\rm aq})}\,{\text{y}}^{-1}$ based on increasing ${\text{CO}}_{2({\rm atm})}$, our results for the OA simulations provide annual linear trends (S) for ${\rm pH}$ ($-\,0.0018\,{\text{y}}^{-1}$), and $\varOmega_{AR}$ ($-\,0.0065\,{\text{y}}^{-1}$), over the entire time range (Table 3).

We calculate ToEs for ${\rm pH}$ and $\varOmega_{AR}$ based on imposition of the calculated slopes and $\sigma$ for the entire time series, as well as partial time series in 11-year increments starting in January 1982. After eliminating the partial year of 1981, 11-year intervals represent 1/3 of the dataset. When calculated over the entire time series using annual averaged data (cf. (Keller et al. 2014)), we find our estimate of ToE for pH in the GOM is longer (approximately 20 years vs 14 years). We speculate that the main reason for this difference is the result of using higher fidelity data to model pH, which imparts greater variance in the ToE estimate.

**Table 5:** Time of emergence in years for ${\rm pH}$ and $\varOmega_{AR}$ based on time series over different time intervals

Time interval	Omega	pH
1981–2014	102.0 (0.33)	35.6 (0.033)
1982–1992	97.5 (0.31)	32.3 (0.029)
1993–2003	103.4 (0.34)	36.0 (0.032)
2004–2014	100.7 (0.32)	28.4 (0.025)

The standard deviation ($\sigma$) of each time series is indicated in parentheses

When based on monthly data and its full variance, longer ToE values are estimated (Table 5). We also find that the ToE for $\varOmega_{AR}$ is considerably longer than for ${\rm pH}$, particularly for time intervals with high variance. Indeed, given the variability seen over the last 34 years, application of Eq. 9 to the data suggests it could take up to a century of observations for an OA signal to emerge in $\varOmega_{AR}$. The differences in ToEs for different intervals are consistent with our findings that variability in ${\rm pH}$ is dominated by the OA signal, while $\varOmega_{AR}$ responds more strongly to perturbations arising from both OA and thermodynamic variability that operates over interannual to decadal timescales. Such results point to difficulties in trend analyses when using inadequately long time series or data of poor temporal resolution that are averaged annually. Further, the ToE estimate used here cannot provide a date at which the data set will reveal that OA is actually dominating the biogeochemical signal, but instead can only indicate the time it would take for the imposed slope to emerge through the variance. For example, trend analyses performed with the last interval of our data set (2004–2014) may conclude that $\varOmega_{AR}$ is steadily increasing, while over the longer time span it is actually decreasing in response to OA.

The fact that the ToEs estimated for the GOM are longer than for than many ocean regions described in Keller et al. (2014) highlights the importance of understanding how local physical and biological processes affect the carbonate system. In our data $\varOmega_{AR}$ shows an annual range between 0.7 and 1.05 in our time series, which is more than twice as large as what is typically observed at open ocean sites such as the Hawaii Ocean Time Series (Doney et al. 2009). The annual cycle of SST and salinity (with its attendant TA change), can account for most of the observed variability (see Fig. 2, top panels). When extreme events like the one experienced over the last decade are added to the seasonality in SST and salinity, the range of $\varOmega_{AR}$ values experienced by calcifying ecosystems becomes even larger. An important finding from these analyses centers on the need for long-term sustained observations in order to establish OA treands and to distinguish the different effects that chemical, physical and biological processes have on the observed signals and trends. Presently such time series are rare, with few (e.g. Hawaiian Ocean Time Series; Bermuda Atlantic Time Series) possessing the required data holdings necessary to resolve the drivers of carbonate system variability.

### Potential significance to ecosystems

Questions arise concerning how rapid changes in the carbonate system may affect calcifying ecosystems. Many organisms have life histories that start in the water column prior to settlement in the benthos, and these early stages may have distinct sensitivities to surface carbonate parameter thresholds (Waldbusser and Salisbury 2014; Waldbusser et al. 2015). For example, Salisbury et al. (2008) have shown slower shell formation and growth in larvae of the commercially harvested GOM clam species *Mercenaria mercenaria* when $\varOmega_{AR}<1.6$.

Sutton et al. (2016) investigated present-day coastal $\varOmega_{AR}$ distributions using ${\rm pH}$ and ${\text{pCO}}_{2({\rm aq})}$ data from several buoyed assets, including the Coastal Western GOM Mooring, located at $43.02^{\circ }{\text{N}}$, $70.54^{\circ }{\text{W}}$. They also modeled preindustrial values, making assumptions about ${\text{pCO}}_{2({\rm atm})}$ values of the past. One finding was that during preindustrial times, $\varOmega_{AR}$ never dropped below the 1.6 threshold at the GOM site. However, in present day conditions the threshold is exceeded in the coastal GOM 11–31% of the time from December through April, with peak exposure to low $\varOmega_{AR}$ in February and March. While the average SSTs during this time are typically below the $11\,^{\circ }{\text{C}}$ necessary to initiate clam spawning (Ropes and Stickney 1965), continued warming in combination with OA could create conditions where larval shellfish are exposed more frequently to suboptimal $\varOmega_{AR}$, leading to less growth. While it is beyond our scope to speculate whether the recent events could have affected ecosystem function, we note that during the last decade, surface ocean ecosystems in the GOM have been exposed to an $\varOmega_{AR}$ range that contains nearly the entire envelope of values observed over the 34-year time series (exposure range since 2004 = 1.2; time series max–min = 1.3). To assess whether an ecosystem or species is at risk or aided by such events, it is important to characterize the drivers and ranges of chemical conditions over periods of low and high variance, and to better account for organismal responses to such conditions.

Our work highlights the importance of long-term monitoring of coastal ecosystems, especially those in ecosystems like the GOM that experience high variability at multiple timescales. Only time series data taken at regular intervals and over decades enable us to overcome signal-to-noise issues, thereby allowing the identification of extreme events and the separation of signals into those affected by physical, biological, and OA processes. Because long-term observation assets are costly to deploy and maintain, it is incumbent upon the ocean carbon modeling communities to continue efforts to resolve variations in the physical processes that control ${\text{CO}}_{2({\rm aq})}$ on timescales from episodic events to decades and longer.
